# Mild Cognitive Impairment: Vascular Risk Factors in Community Elderly in Four Cities of Hebei Province, China

**DOI:** 10.1371/journal.pone.0124566

**Published:** 2015-05-11

**Authors:** Yumei Wang, Mei Song, Lulu Yu, Lan Wang, Cuixia An, Shunjiang Xun, Xiaochuan Zhao, Yuanyuan Gao, Xueyi Wang

**Affiliations:** 1 Department of Psychiatry, First Hospital of Hebei Medical University, Shijiazhuang, 050031, China; 2 Mental Health Institute of Hebei Medical University, Shijiazhuang, 050031, China; 3 Brain Aging and Cognitive Neuroscience Laboratory of Hebei Medical University, Shijiazhuang, 050031, China; Banner Alzheimer's Institute, UNITED STATES

## Abstract

**Background:**

Evidence has demonstrated that vascular risk factors (VRFs) contribute to mild cognitive impairment (MCI) in the elderly population. Because of the race and different diagnosis standard, there is still no definitive conclusions.

**Objective:**

To estimate the VRFs and potential protective factors for MCI in elderly population living in the community in North China.

**Methods:**

A total of 3136 participants entered the study. They were screened for hypertension, coronary heart disease (CHD), and cerebrovascular disease (CVD). Cognitive function was assessed with Mini-Mental State Examination (MMSE) and the Montreal Cognitive Assessment (MoCA). The diagnosis of MCI was made according to Petersen’s criteria. We investigated the relationship between vascular risk factors, potential protective factors and MCI.

**Results:**

A total of 2511 (80%) participant belonged to normal group and 625 (20%) participants showed MCI. Multiple logistic regression analysis demonstrated that stroke and diabetes, but not hypertension or CHD was associated with MCI. Besides, exercise habit could lower the risk of MCI.

**Conclusions:**

Vascular Risk Factors, including stroke and diabetes, rather than hypertension and CHD are independent risk factors of MCI. Involvement in physical activities seems to reduce the risk of MCI.

## Introduction

Mild cognitive impairment (MCI) is an intermediate stage between the expected cognitive decline of normal aging and the more serious decline of dementia. It is a common clinical manifestation affecting the aged people. The progression rates from MCI to dementia is suggested range between 5.4% and 11.7% per year; moreover, patients with MCI have higher risk of progression to dementia compared with healthy controls [1,2]. The cause of MCI is unknown, however MCI and dementia such as Alzheimer's disease (AD) have some similar risk factors, such as heart problems, blood pressure and diabetes [3]. At present, the number of people affected by dementia is continually increasing. It is estimated that to date 24 million people have dementia [4]. Nowadays, the field of aging and dementia is moving toward an earlier identification of clinical impairment, and MCI has attracted more and more attention recently. MCI involve problems with memory, language, thought, and judgment that are greater than normal age-related changes, which are not significantly enough to interfere with daily activities [5]. However, there are no modified treatments for dementia so far. The early intervention of cognitive impairment appears to be very important for the prevention of dementia. Cerebral hypoperfusion can contribute to structural and functional changes in the brain, which ultimately leads to cognitive impairment [6]. Hypertension, coronary heart disease (CHD), and stroke (including cerebral infarction and cerebral hemorrhage) may be potential risk factors for MCI, by affecting cerebral hypoperfusion. However, the results are not consistent, some research suggest that there are no associations between hypertension and MCI [7]. There are still no definitive conclusions about the relation between vascular risk (VRFs) and MCI based on published data [8]. Moreover, data on the correlation between VRFs and MCI are still in the absence of Chinese population. The propotion of elderly people has been increasing in China. So China may have the largest number of elderly people with dementia in the world. Therefore, this issue needs to be further investigated in China given race and regional differences. Besides, some studies also demonstrate that the treatment of VRFs can slow down the progression of cognitive impairment and dementia [9]. Exploring the concerning factors are important for primary prevention and early intervention. Besides, several surveys have been conducted elderly Chinese people, in which only Mini-Mental State Examination (MMSE) was used as the criterion for MCI[10].

The present study aimed to investigate the association of MCI with several VRFs and potential protective factors for MCI in the elderly population in North China, using a series of neuropsychological scales.

## Methods

### Study Participants

The sampling design and diagnosis criteria could be found in our previous study[11]. We briefly provide them again in the present study. The cross-sectional population-based study was conducted in Hebei province between January 2010 and January 2011. The sample size was calculated on the basis of an expected prevalence of MCI[12]. A multistage stratified cluster sampling method with selection probabilities proportional to size was designed to recruit participants, based on the urban resident registry of the Hebei province, 2010 (Sixth Nationwide Population Census in China, 2010). Considering possible differences in the population, such as rural lifestyle, education status, and other demographic factors, we selected the samples from community in city. A multistage stratified cluster sampling method with selection probabilities proportional to size was designed to recruit participants. A four-stage sampling process was performed for this study, which used simple random sampling at each stage. In the first stage, 4 out of 11 cities were selected randomly in the Hebei province, China. In the second stage, 12 districts were selected randomly from the 4 cities. In the third stage, 36 communities were selected randomly from the 12 districts. In the fourth stage, stratified by age and gender, 90 residents aged 60 year or older in a cluster were selected in each community. Individuals volunteered from clinic of community service center. The inclusion criteria were as follows: (1) 60 years and older; (2) no history of neuropsychiatric disorders; (3) no evidence of neoplastic diseases; (4) agreed to participate in the study. Exclusion criteria were as follows: (1) acute stress state, for example, acute medical disorders; (2) diagnosed with dementia, clinical diagnosis of dementia was based on the DSM-IV criteria; (3) severe organ dysfunction; (4) the participants or the guardians refused to participate in the study. At last, a large cohort of 3,136 aged participants entered the study. Community-based individuals volunteered from clinic of community service center.

### Ethics Statement

Our study was authorized by the Ethics Committee of the First Hospital of Hebei Medical University and all participants gave written informed consent.

### Study Design and Measurements

This study was divided into two steps: a screening stage for all the eligible participants and a second stage for the confirmation of the suspected cases of MCI.

In the step1, the 3136 eligible participants took part in a face-to-face interview with a standardized questionnaire. The interview was conducted by trained graduate students and mental health clinicians. The demographic information and history of hypertension, diabetes, stroke and coronary heart disease were collected. The instrument applied to screen for cognitive impairment was Mini-Mental State Examination (MMSE) [12] and the Montreal Cognitive Assessment (MoCA)[13]. The MMSE has become one of the most widely used cognitive screening instruments for MCI, which covers various areas of cognitive domains. The MoCA was designed as a rapid screening instrument for cognitive dysfunction. It was developed in response to the poor sensitivity of the MMSE in distinguishing clients with cognitive impairment from normal elderly. The score of the MMSE (≤27) and that of the MoCA (<26) were taken together to evaluate cognitive impairment[14].

In the step 2, the following neuropsychological tests administered by psychologists were used for the evaluation for different cognitive function domains: Wechsler Memory Scale-Revised is a neuropsychological test designed to measure different memory functions, such as logical memory, associative learning, visual recognition and picture recall [13]; Wechsler Adult Intelligence scale-Chinese revision and Trail Making Test provide information on visual attention, scanning, speed of processing, mental flexibility, and executive functions[15,16] and Activity of Daily Living scale is an appropriate instrument to detect problems in performance activities of daily living [17]. The MMSE and MoCA are typically used to assess global cognition functioning. Different neurological test variables contributed to the diagnosis of MCI. Besides, the Center for Epidemiology Scale-Depression (CES-D) survey was used to assess the frequency of depressive symptoms during the past week.

### Diagnosis of MCI

We have assessed the cognitive impairment in 3,136 subjects (1,275 males and 1,861 females). A cross-sectional cohort study was employed. For the evaluation of MCI, the diagnostic criteria proposed by Petersen er al.[14] were applied (Table 1): (1)memory complaint; (2) Activities of Daily Living scale score <26 (20 items); (3) normal general cognitive function; A: MMSE score between 20 and 27 [cutoff points for illiterate (≤ 20), primary school (≤ 23) and secondary school and above (≤ 27)]; B: MoCA score <26; (4) subjects having a score 1.5 SD below the cut off were diagnosed as having MCI if they also had a score of 0–0.5 on the Clinical Dementia Rating scale; (5) no dementia; clinical diagnosis of dementia was based on the DSM-IV. Although MCI was categorized according to the subtypes defined by Petersen, for the purposes of analyses, participants with all subtypes of MCI were grouped together. The cognitive and functional status of all patients was assessed by using Activity of Daily Living scale (ADL), Mini-Mental State Examination (MMSE) and Montreal Cognitive Assessment (MoCA), and recruited participants were divided into normal control and the MCI group.

**Table 1:** Diagnostic criteria for MCI according to Petersen.

a Memory complaint by participant or family
b Normal activities of daily living
c Normal general cognitive function
d Objective impairment in one area of cognitive function as
evidenced by scores of >1.5 SD of age-appropriate norms (b)
or abnormal memory function for age (c)
e No dementia

### Neuropsychological Tests and Personal Information questionnaire

A questionnaire was constructed to collect personal information and demographic information, including age, gender, medical history, level of formal education, occupation, marital status, smoking status, and alcohol consumption. The survey was performed by professional psychiatrist via face-to-face interview in hospital or community service center. Medical history included coronary heart disease (CHD), hypertension, cerebral infraction, cerebral hemorrhage and diabetes. The diagnosis of CHD fulfilled defined as a history of silent myocardial ischemia, angina pectoris, myocardial infarction, or ischemic cardiomyopathy. Cerebrovascular disease was identified as of a history of cerebral infraction, cerebral hemorrhage.

### Statistical Analysis

The estimations of the means and proportions were calculated to describe the demographic characteristics, hypertension, CHD and stroke history. Proportions were compared using χ2. test. *t*-test was used to compare group differences in case of continuous variables. Related factors of MCI were analyzed using multivariate logistic regression analyses, from which the odds ratios (OR) and 95% confidence intervals (CI). The analyses of all data were performed using SAS software, version 9.1. Statistically significant findings were determined using a 2-tailed *P* value of 0.05.

## Results

### Demographic Characteristics

We recruited 3,136 subjects (1,275 males and 1,861 females) from four districts each in the cities of Shijiazhuang, Tangshan, Zhangjiakou, and Handan in Hebei province, China. Table 2 shows the characteristics of the 3,136 participants, The average age was 69.3 ± 6.8 years (range, 60–94 years), 1,275 (40.1%) were males; the mean age of the participants was 69.1 ± 6.8 years (range 60–94 years); 29.6% were in the 60- to 64-year age group; 47.5% had engaged in blue-collar work; 85.2% of the participants have the habit of exercise. 81.7% were currently married. 18.9% of the participants had coronary heart disease, 45.6% had hypertension and 10.3% had stroke.

**Table 2:** Demographic charagteristics of the study population.

Characteristics	N	%
Age group		
60–64 years	928	29.6
65–69 years	724	23.1
70–74 years	766	24.4
75–79 years	464	14.8
≥80 years	257	8.1
Gender		
Male	1275	40.7
Female	1861	59.3
Marital status		
Never married	10	0.3
Married	2543	81.7
Windowed	560	18.9
Occupation		
No work	215	7.0
blue-collar work	1467	47.5
White-collar worker	1405	45.5
Exercise		
Yes	2655	85.2
No	462	14.8
Coronary heart disease		
Yes	560	18.9
No	2391	8.1
Hypertension		
Yes	1394	45.6
No	1651	54.4
Cerebral fraction		
Yes	278	10.4
No	2808	89.6
Cerebral hemorrhage		
Yes	45	3.3
No	3058	96.7
Stroke		
Yes	319	10.3
No	2787	89.7
CES-D		
Yes	237	7.6
No	2892	92.4
Diabetes		
Yes	480	15.7
No	2753	84.3

### Predictors of MCI by univariate analysis

A total of 2,511(80%) participants belonged to the normal group and 625 (20%) participants showed MCI according to the Petersen’s criteria. Table 3 shows the univariate analyses of selected demographic variables for MCI. Blue-collar occupation was associated with a higher prevalence than white-collar occupation (P<0.0001). The prevalence of MCI was higher in participants without exercise than with exercise habit (P<0.0001). Marital will affect the prevalence of MCI (P<0.0001). Stroke (including cerebral infraction or cerebral hemorrhage) history could predict MCI, however, CHD and hypertension could not predict the MCI.

**Table 3:** Univariate analysis of cognitive impairment.

Variables	Cognitively impairment	Cognitively normal	X ^2^	p value
	n(%)	n(%)		
Gender				
Male	237(37.9)	1038(41.3)	2.42	0.12
Female	388(62.1)	1473(58.7)		
Age group			231	<0.0001*
60–64 years	87(13.9)	841(33.5)		
65–69 years	112(17.9)	612(24.4)		
70–74 years	165(26.4)	601(23.9)		
75–79 years	137(21.9)	327(13.0)		
≥80 years	124(19.8)	133(5.2)		
Marital status			50.2	<0.0001*
Never married	4(0.6)	6(0.2)		
Married	445(71.9)	2098(84.2)		
Windowed	170(27.5)	390(15.6)		
Occupation			26.2	<0.0001*
No work	59(9.6)	156(6.3)		
Blue-collar work	330(53.5)	1137(46.0)		
White-collar work	227(36.9)	1178(47.7)		
Exercise			52.8	<0.0001^#^
Yes	473(75.9)	2182(87.5)		
No	150(24.1)	312(12.5)		
Hypertension			0.16	0.69
Yes	279(46.5)	1115(45.6)		
No	321(53.5)	1330(54.4)		
Stroke			16.4	<0.0001*
Yes	521(85.3)	2266(90.8)		
No	90(14.7)	229(9.2)		
Coronary Heart Disease			0	1
Yes	108(19)	452(19)		
No	461(81)	1930(81)		
CES-D			52.5	<0.0001*
Yes	521(69.5)	90(3.7)		
No	229(30.5)	2359(96.3)		
Diabetes			7.56	0.006*
Yes	117(19.4)	363(14.8)		
No	487(80.6)	2086(85.2)		

*P < 0.01

#### Predictors of MCI by multiple logistic regression

Multivariate logistic regression analysis found that lack of exercise habit(OR = 2.0, 95%CI = 1.6–2.6), stroke history (OR = 1.4, 95%CI = 1.0–1.9), diabetes (OR = 1.4, 95%CI = 1.04–1.79) were correlated with higher prevalence odds for MCI (Table 4). Hypertension and CHD were not associated with MCI.

**Table 4:** Multivariable logistic regression analysis of cognition impairment.

Variables	Risk factors	OR	95%CI
Occupation			
	No work	1	
	Blue collar work	0.4	0.1–1.6
	White collar work	0.2	0.04–0.9
Exercise			
	Yes	1	
	No	2.0	1.6–2.6
Stroke			
	No	1	
	Yes	1.4	1.03–1.9
Diabetes			
	No	1	
	Yes	1.4	1.04–1.79

### Comparing neuropsychological Test Score between two groups

Mean neuropsychological test scores are listed in Table 5. The *t*-test was used to compare cognition function between cognitively impairement group and cognitive normal group. The two groups were significantly different in cognitive score.

**Table 5:** Neuropsychological Test Score of cognitively impairment group and cognitive normal group.

Neuropsychological Test	Cognitively impairment (n = 625)(mean±std)	Cognitively normal (n = 2511)(mean±std)	t	p value
Mini-Mental State Examination	25.9±5.1	28.3±3.9	12.9	<0.001
Montreal Cognitive Assessment	23.1±5.2	27.2±5.9	16.1	<0.001
Activity of Daily Living	27.2±12.7	21.1±4.5	18.2	<0.001
Wechsler Memory Scale-Revised				
Verbal Paired Associates	7.7±3.9	9.7±3.3	13.1	<0.001
Visual Reproduction	9.5±3.7	11.2±3.2	11.0	<0.001
Immediate memory	5.2±3.3	6.6±2.6	11.8	<0.001
Picture recall	13.3±4.2	16.0±3.5	16.1	<0.001
Wechsler Adult Intelligence scale-Chinese revision				
Similarities	13.9±5.3	17.5±4.3	17.6	<0.001
Arithmetic	9.5±3.4	12.1±3.4	16.7	<0.001
Digit Symbol-Coding	28.8±11.6	34.8±9.2	13.7	<0.001
Picture Completion	8.4±2.4	9.4±1.3	14.6	<0.001
Block Design (s)	91.3±42.0	74.8±22.6	13.3	<0.001
Vocabulary	15.0±4.0	16.6±5.0	6.9	<0.001

## Discussion

The present study has shown that stroke (cerebral hemorrhage or cerebral infarction), but not hypertension and coronary heart disease (CHD), are associated with MCI in Chinese elderly. Mild cognitive impairment is more likely to present in patients with cerebral vascular disease than those without it. 20% of the subjects had MCI, suggesting 1 of 5 individuals have MCI. The result is higher than most studies of MCI prevalence in China as a meta-analysis study (12.7%)[18] and a community-dwelling residents study (15.7%)[19]. While reviews report the prevalence of MCI as ranging from 5% to 29%[20,21] in worldwide studies. Nie et al. indicate that female is more dangerous than male in getting the MCI[18]. In the present study, female percentage (59.3%) was higher than male percentage (40.7%). Besides, at each 5-years increase in age, the prevalence of mild cognitive impairments increased 1.27,1.45,1.39 and 1.35 times[18]. 70.4% participantes are aged over 65 years in our study. The above reasons may lead to the higher prevelence of MCI than previous studies in China, thus we should carefully apply the results to other samples.

Cardiovascular risk factors including hypertension have been shown to increase the risk of Alzheimer's disease and individuals with hypertension are more susceptible to increased cognitive decline [22]. Some studies indicate that only higher diastolic blood pressure increases the risk of cognition impairment [23]. Some studies suggest that only midlife high blood pressure is a risk factor for late-life cognitive impairment and dementia [24]. So, the result about the association between hypertension and cognitive impairment is not coinciding. We found no relationship between the hypertension and mild cognitive impairment. There are two possible explanations for the present results. First, increasing evidence has suggested that antihypertensive agents have a decreased risk of dementia in individuals with hypertension and antihypertensive treatments need to be carried out in early stage[25], for example, ACEI and Nilvadipine could improve the cerebral blood flow, improve memory and executive function. In our study, 86.4% participants are receiving medicine treatment for hypertension or coronary heart disease. Efforts to lower blood pressure (BP) might promote reversion from mild cognitive impairment and normal cognitive function[26].Second, one study suggests that it is blood pressure fluctuations but not average BP were significantly with cognitive impairment in a long-term study[27], BP fluctuation but not average BP may accelerate the artery atherosclerosis.

Our study suggests that CHD is not associated with MCI. Some studies have found no significant contribution of CHD to cognitive impairment[28], for example, one study suggest that heart diseases diagnosed at midlife did not increase the risk of later dementia and AD and only late-life heart diseases increase the subsequent risk of dementia and AD [29]. Ganguli et al suggest only heart failure, but not all CHD is a risk factors for MCI [7]. However, several studies found that CHD is associated with cognitive decline, such as verbal memory, executive function and the global cognition. Therefore, the relationship between CHD and MCI is still contradictory. According to our results, CHD could not contribute to cognitive impairment. In the present study, we used MMSE, MoCA and ADL to assess global cognitive function to make the diagnosis of MCI. In other words, we did not only evaluate the memory complaint and general cognitive function, but also the activities of Daily Living. The stringent criteria were made for participant inclusion, which may lead to older participants with cognitive decline entering the study, but who were not suitable for the present study. So, different neurological test variables contributed to the diagnosis of MCI. Besides, we only collected the information about CHD history, not care for the CHD duration, diagnosis the CHD in midlife or late-life. Therefore, we need to further collect the CHD history.

Cerebrovascular disease (CVD), especially stroke including cerebral hemorrhage and cerebral infarction, increases the risk of both MCI and AD [7,30]. Ischemic stroke and Alzheimer's disease (AD), despite being distinct disease entities, share numerous pathophysiological mechanisms such as those mediated by inflammation, immune exhaustion, and neurovascular unit compromise, for example PKC (protein kinase C)-dependent events and β-amyloid production may serve as an important connection linking age-related brain injury [31]. One study shows a strong relationship between ESR (erythrocyte sedimentation rate) and hippocampus volume, as well as with cognitive performance among post stroke patients [32]. Cerebrovascular diseases consists different types, variant CVD subtype shows different risk for MCI. The prevalence of cognitive impairment is higher in vessel occlusion than that in lacuna infraction three month after stroke [33]. Consistent with the most findings, our present study demonstrated that stroke was associated with MCI. Patients with low cognitive level before stroke function are at high risk of dementia over time [34], therefore we should follow them closely for further cognitive decline. History of CAD may be a surrogate marker for clinically significant atherosclerosis, which also affects the brain structural. Prevention of recurrent strokes is obviously crucial to reducing the burden of cognitive decline and dementia after stroke. Modern imaging and analysis techniques such as 7T MRI and PET will help to elucidate the mechanism of the post stroke disease [33], which we help us make a combination therapy to target multiple components of post stroke disease.

Our results showed that, with increasing age, the prevalence of MCI increased, which is consistent with previous result [35]. One study suggested that occupational status may be related to brain aging [36]. Our study found that people with blue-collar work were more likely to have MCI, which is consistent with previous studies [37–39]. Our study also suggested that married people are less likely to suffer from cognitive impairment than those who are divorced or windowed. One study shows that marital status has positive impacts on memory across adults and old age [40], which suggest cognitive stimulation offered by a partner may protect the brain from deterioration.

The results of present study indicate that the cognitively normal elderly population had more exercise habit than the group with MCI. These results were consistent with those of previous studies, which found that waking, running, dancing, Tai chi or other physical activities may have effect in decreasing the probabilities of developing MCI [41].

We have to acknowledge a few limitations in this study. First, this is only a cross-sectional study. The absence of follow-up data becomes more pressing in order to establish the validity of the MCI diagnosis used here. In addition, the diagnostic criteria of MCI are in the developmental stages and require further validation. Second, we only collected the information whether subjects with hypertension, stroke and /or coronary heart disease are using drug (86.4%) and we did not collect the information about detailed medication. In other words, the use of drug to control hypertension and/or CHD is incomplete. Eventually, we could not find the correlation between the hypertension or CHD history and MCI in the study. Third, there is a selection bias in the study. To minimize the selection bias, we used the whole population of the four selected cities to do the weighting adjustments.Based the above reasons, we should carefully apply the results to other samples.

## Conclusion

Stroke including cerebral hemorrhage or cerebral infarction and diabetes, but not hypertension and CHD are independent risk factors of MCI. Involvement in physical activities seems to reduce the risk of MCI. The intervention to patients with stroke and/or diabetes may contribute to normal cognition and a healthy brain. Identified these factors may allow elderly population to make better choices and provide information for clinical practices.
